# Learning Meta-Learning (LML) dataset: Survey data of meta-learning parameters

**DOI:** 10.1016/j.dib.2023.109777

**Published:** 2023-11-07

**Authors:** Sonia Corraya, Shamim Al Mamun, M. Shamim Kaiser

**Affiliations:** Institute of Information Technology, Jahangirnagar University, Bangladesh

**Keywords:** Multiple Intelligence, Learners’ biosocial parameter, Chronotype, Imposter phenomenon, Illusion of competence

## Abstract

The ‘Learning Meta-Learning’ dataset presented in this paper contains both categorical and continuous data of adult learners for 7 meta-learning parameters: age, gender, degree of illusion of competence, sleep duration, chronotype, experience of the imposter phenomenon, and multiple intelligences. Convenience sampling and Simple Random Sampling methods are used to structure the anonymous online survey data collection voluntarily for LML dataset creation. The responses from the 54 survey questionnaires contain raw data from 1021 current students from 11 universities in Bangladesh. The entire dataset is stored in an excel file and the entire questionnaire is accessible at (10.5281/zenodo.8112213)

In this article mean and standard deviation for the participant's baseline attributes are given for scale parameters, and frequency and percentage are calculated for categorical parameters. Academic curriculum, courses as well as professional training materials can be reviewed and redesigned with a focus on the diversity of learners. How the designed courses will be learned by learners along with how they will be taught is a significant point for education in any discipline. As the survey questionnaires are set for adult learners and only current university students have participated in this survey, this dataset is appropriate for study andragogy and heutagogy but not pedagogy.

Specifications Table

**Table:** 

Subject	Data Science
Specific subject area	Meta-learning parameters, Multiple Intelligence, Chronotype
Data format	Raw
Type of data	Excel file
Data collection	Data are collected through an online anonymous volunteered survey. Established and validated questions are adapted for most of the parameters in this survey which are explained with respective source references later in this article. A structured survey questionnaire with Bangla translation was made available to the participants through an online link. (See “questionnaire” file at 10.5281/zenodo.8112213). Convenience sampling and Simple Random Sampling methods are incorporated to structure the survey. Data were collected from July 2022 to February 2023. Prior to accessing the survey, participants were presented with an information consent form and only those who agreed proceeded to answer the survey. The Inclusion criteria for survey participants are: i. Currently studying at any university in Bangladesh ii. The minimum age is 18 years old
Data source location	Data were collected from 11 different universities in Bangladesh.
Data accessibility	Repository name: zenodo Data identification number: 10.5281/zenodo.8112213 Direct URL to data: 10.5281/zenodo.8112213 Instructions for accessing these data: The dataset presented in this article is open for public access. It is mandatory to follow the correct citation guidelines when using this LML dataset.

## 1 Value of the Data

• These data can be useful to understand the association among the meta-learning factors of adult learners.
• Research findings with this data will help education designer, adult self-learners and learners’ meta-data analysts.
• Academic education and industrial training program structure might be reviewed based on the analysis findings with this data, to raise the quality of learners’ accomplishments.
• Underlying patterns of this dataset can direct significant insights about learning trends and the natural grouping of the learners.
• The data collection can be replicated in other countries or with other kinds of meta-learning parameters to make a comparison between them.

## 2 Data Description

This data article reports survey questionnaire [14], raw data [14], and baseline characteristics of the study participants (see Table 1). In Table 1, categorical data are reported as frequency (percentages), and continuous data as mean (standard deviation). LML dataset contain nominal, ordinal, and continuous variables.

**Table 1:** Baseline characteristics of the survey participants.

Characteristic	Value
Number of participants, n	1021
Age (years), mean (SD)	21.72 (1.76)
Gender, n (%)	
Male	638 (62.49)
Female	383 (37.51)
Degree of Illusion of Competence, n (%)	
Mild	241 (23.60)
Moderate	465 (45.54)
Severe	315 (30.85)
Sleep duration (hours), mean (SD)	6.29 (1.14)
Chronotype, n (%)	
Morningness	313 (30.66)
Intermediate	369 (36.14)
Eveningness	339 (33.20)
Experience of Imposter Phenomenon, n (%)	
Few	32 (3.13)
Moderate	324 (31.73)
Frequent	557 (54.55)
Often and intense	108 (10.58)
Multiple Intelligence, mean (SD)	
Linguistic	14.12 (2.93)
Logical-Mathematical	15.83 (2.59)
Spatial-Visual	12.91 (2.93)
Bodily-Kinesthetic	12.58 (2.79)
Musical	13.09 (3.21)
Interpersonal	13.98 (3.11)
Intrapersonal	14.91 (2.73)

## 3 Experimental Design, Materials and Methods

### 3.1 Sampling

As per the 47^th^ Annual Report 2020 (Year of Publication: October 2021) of University Grants Commission of Bangladesh [5], the total number of students at both public (including affiliated and constituent colleges/madrasas) and private universities is 46,90,876. It was unfeasible to conduct probability sampling on this huge population. For this, Simple Random Sampling (SRS) with Convenience sampling is incorporated for data collection to create this LML dataset. In this SRS and Convenience sampling structure, all universities, classrooms, and students are chosen randomly with convenience. Among the 11 universities, there were 4 general public universities, 4 general private universities, 2 engineering and technology universities and 1 institute of engineering and research. Required standard Sample Size (SS) to represent the population is calculated using Slovin's Formula [6,7]. SS can be represented as below:

$$\text{SS}=P/(1+P*C^{2})$$

where, P is the known population size and C is the margin of error [6,7].

SS for LML dataset with P= 46,90,876 and C=0.05 with 95% confidence interval is 400.

The sample for the survey consisted of adult (age>18) [13] participant who were approached verbally in the classroom and invited to voluntarily participate. Before accessing the survey, participants were provided with an information consent form, and only those who agreed proceeded to answer the survey. As the required standard SS to represent the population is 400, with an expectation that 30% of the people invited to take the survey will actually respond, we planned to approach (400/30% = 1333.33 ≈) 1300 to 1400 students (approx.). We ended up with 1021 responses, from which it can be said the survey response rate is (1021/1300 to 1021/1400 =) 78.54% to 72.92%. An exact response rate (or completion rate) cannot be specified for this survey as all the students were approached only verbally in person in the classrooms and were invited to voluntarily participate then or later after class.

Survey questionnaires were made available to the participants through an online Google form link. The inclusion criteria for survey participants are:

i. Currently studying at any university in Bangladesh
ii. The minimum age is 18 years old

### 3.2 Survey Questionnaires

In the survey questionnaires, for most of the parameters, established and validated questions are adapted. The purpose of the LML dataset is to provide naturalistic self-reported data that is assumed to be linked with individuals’ general learning processes. Meta-learning parameter wise survey questionnaires are explained below.

**Fig. 1:**
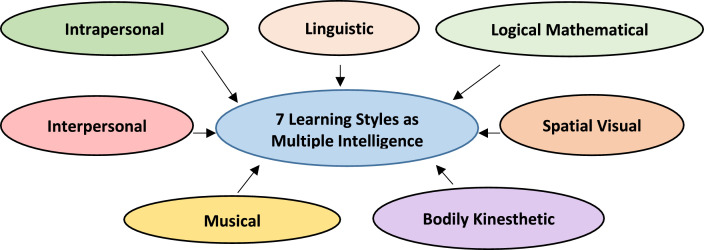
The 7 learning styles as multiple intelligence.

• Age (Question 2): Participants selected a single category that represented their age. The minimum age option in the survey was 18 years, and the maximum age option was above 26 years.
• Gender (Question 3): Participants selected one option as their gender identity: “Male,” “Female,” or “Other/Non-binary”.
• Degree of Illusion of Competence (Question 4): Participants rated their own degree of ‘Illusion of Competence’ experience as “mild”, “moderate”, or “severe”. To the best of our knowledge, there exists no validated question for ‘Illusion of Competence’ measurement. The measures used here were developed for the creation of the LML dataset.
• Sleep duration (Question 5): University students fall under the inclusive sleep range of 6–11 hours [8]. The survey participants had a single selection choice for each of the sleep hours in this range.
• Chronotype (Questions 6–10): Each of the 5 questions for measuring chronotype had different single-selection options. Information on interpreting the scores is available in the data dictionary of the LML dataset file [14]. Questions were taken from the reduced Morningness-Eveningness Questionnaire (rMEQ) [10] for identifying the chronotype of individuals. Only questions 1, 7, 10, 18, and 19 of the original Morningness-Eveningness Questionnaire [9] are included in the rMEQ [10].
• Experience of Imposter Phenomenon (Questions 11-20): Participants provided their answers regarding their own experience of the ‘Imposter Phenomenon’ on a 5-point Likert scale ranging from 1 (‘Not at all true’) to 5 (‘Very true’). The data dictionary of the LML dataset file [14] contains details on how to interpret the final scores. The selected 10 question numbers from the Clance Imposter Phenomenon questionnaire [11] considering redundancy and relevancy for measuring learning handicap-Imposter Phenomenon, are: 1, 2, 5, 6, 7, 11, 12, 15, 19 and 20.
• Multiple Intelligence (Questions 21–55): For each of the 7 sub-intelligences of multiple intelligences, as shown in Fig. 1, there were 5 questions in the survey, and scores were taken on a 4-point Likert scale ranging from 1 (‘Not at all true’) to 4 (‘Very true’). The LML dataset file's [14] data dictionary contains information on how to interpret the scores. After tracing the literature of multiple intelligences [2,4] and looking at the previous relevant studies [3], the scale and questionnaires developed by Chislett and Chapman (2005-2006) [1] have been adapted.

## Limitations

The sample population of the LML dataset has a female to male ratio of (37.51/62.49=) 0.60, which at first glance appears to be biased on gender. However, when taking into account the following ratios, this LML dataset is actually a true representation of the Gender Parity Index, a socioeconomic index used to measure how equally both men and women have access to education. The female to male ratio in Bangladesh's tertiary education was (22.8/27.3=) 0.83 in 2021, according to UNESCO [12]. According to the University Grants Commission of Bangladesh's 47^th^ Annual Report 2020 (Year of Publication: October 2021), the ratio of female to male university students in Bangladesh is (43/57=) 0.75 [5].

As learners, only current students from 11 different universities in Bangladesh were surveyed using SRS and Convenience sampling. In the future, the dataset will be expanded by including a wider range of learners from vocational training institutes, music schools, art schools, special education, and more.

## Ethics Statement

Ethical approval [Ref No. BBEC,JU/M 2022/01 (18)] has been obtained from the Biosafety, Biosecurity and Ethical Clearance Committee, Jahangirnagar University. The declaration of Helsinki was not mandatory for this non-medical dataset.

## CRediT authorship contribution statement

**Sonia Corraya:** Conceptualization, Methodology, Data curation, Visualization, Writing – original draft. **Shamim Al Mamun:** Writing – review & editing, Supervision. **M. Shamim Kaiser:** Writing – review & editing, Supervision.

## Data Availability

Learning Meta-Learning (LML) Dataset: Survey Data of Meta-Learning Parameters (Original data)
